# A comprehensive prognostic score for head and neck squamous cancer driver genes and phenotype traits

**DOI:** 10.1007/s12672-023-00796-y

**Published:** 2023-10-28

**Authors:** Wen Zeng, Fangfang Xie, Yiyun Pan, Zhengcong Chen, Hailong Chen, Xiaomei Liu, Keqiang Tian, Dechang Xu

**Affiliations:** 1 Ganzhou Cancer Hospital, Gannan Medical College Affiliated Cancer Hospital, No.19, Huayuan Road, Zhanggong Avenue, Ganzhou, Jiangxi People’s Republic of China; 2 Ganzhou People’s Hospital, Ganzhou, 341000 Jiangxi People’s Republic of China

**Keywords:** Head and neck cancer, Prognosis, Transcriptomics, Cancer genes

## Abstract

**Background:**

Head and neck squamous cancer (HNSCC) presents variable phenotype and progression features. Clinically applicable, high-accuracy multifactorial prognostic models for HNSCC survival outcomes are warranted and an active area of research. This study aimed to construct a comprehensive prognostic tool for HNSCC overall survival by integrating cancer driver genes with tumor clinical and phenotype information.

**Methods:**

Key overall survival-related cancer driver genes were screened from among main effector and reciprocal gene pairs using TCGA data using univariate Cox proportional hazard regression analysis. Independent validation was performed using the GSE41613 dataset. The main effector genes among these were selected using LASSO regression and transcriptome score modeling was performed using multivariate Cox regression followed by validation analysis of the prognostic score. Next, multivariate Cox regression analysis was performed using the transcriptome score combined with age, grade, gender, and stage. An ‘Accurate Prediction Model of HNSCC Overall Survival Score’ (APMHO) was computed and validated. Enriched functional pathways, gene mutational landscape, immune cell infiltration, and immunotherapy sensitivity markers associated with high and low APMHO scores were analyzed.

**Results:**

Screening 107 overall survival-related cancer genes and 402 interacting gene pairs, 6 genes: CRLF2, HSP90AA1, MAP2K1, PAFAH1B2, MYCL and SET genes, were identified and a transcriptional score was obtained. Age, stage and transcriptional score were found to be significant predictors in Cox regression analysis and used to construct a final APMHO model showing an AUC > 0.65 and validated. Transcriptional score, age, pathologic_N, pathologic_T, stage, and TCGA_subtype were significantly different in distribution between high and low APMHO groups. High APMHO samples showed significantly higher mutation rate, enriched tumor-related pathways including Hypoxia, unfold_protein_response, Glycolysis, and mTORC1 signaling, along with differences in immune cell infiltration and immune checkpoint, interferon-γ pathway and m6A regulator expression patterns.

**Conclusion:**

The APMHO score combining transcriptional and clinical variables showed good prognostic ability for HNSCC overall survival outcomes and was associated with different patterns of phenotypical features, immune and mutational landscape, and immunotherapy sensitivity marker expression. Future studies should validate this score in independent clinical cohorts.

**Supplementary Information:**

The online version contains supplementary material available at 10.1007/s12672-023-00796-y.

## Introduction

In 2018, head and neck cancer was the seventh most common cancer worldwide, with 1,918,030 new cases and 609,360 deaths, accounting for 2.8% (54,000 cases) of all cancers and a little more than 1.8% (11,230 cases) of all cancer deaths in the United States [1, 2]. The occurrence of head and neck cancers is associated with heavy use of tobacco and alcohol, and the current decrease in tobacco-related incidence in global cases is partly related to the decrease in smoking [3, 4]. On the other hand, cases of HPV-associated oropharyngeal cancer induced by HPV type 16 are increasing, with the proportion of HPV-positive oropharyngeal cancers of the head and neck in the United States rising from 16.3% in the 1980s to more than 72.7% in the 2000s and exceeding 72.7% in 2000 [5]. With the rapid ageing of the population and the consequent increase in disease burden, treatment of HNSCCs has become critical. Genetic aberrations are also considered to be major causative factors in carcinogenesis, including epigenetic regulators affecting the tumor microenvironment, the demethylases ALKBH5, YTHDF1, IGF2BP2, and YTHDC2 [6–8]. The evolving understanding of the genetic status of head and neck squamous cell carcinoma has contributed to the revision of the disease classification and management.

As a general diagnostic term, head and neck squamous carcinoma includes a high degree of heterogeneity in clinical features, various survival outcomes, and treatment response [9]. The heterogeneity of head and neck squamous carcinoma includes variations in immunophenotypic, cytogenetic, and molecular features, which pose challenges for predictable residual disease detection and recurrence prediction [10]. The correlation between genetic and phenotypic heterogeneity in head and neck squamous carcinoma is an ongoing research topic, with precision medicine approaches sought to improve treatment outcomes through the combination of appropriate immunotherapy, chemotherapy, and other targeted agents [11]. Recurrence remains the leading cause of death associated with head and neck squamous carcinoma due to the refractory nature of the recurrent disease [12]. Advances in cost-effective high throughput sequencing and machine learning algorithms have enabled large-scale transcriptomic profiling of cancers and the identification of tumor molecular characteristics that can be leveraged clinically for precision management modalities. Several recent clinical trials have demonstrated the clinical translation of transcriptomic feature-based management algorithms for improving treatment outcomes in cancer [13–15]. Transcriptome-based multigene predictive or prognostic panels have been developed for clinical applications in several cancer types [16].

Previous studies have reported transcriptional signatures and gene clusters that predict the risk of recurrence associated with specific clinical subgroups of head and neck squamous carcinoma [17–19], with varying levels of accuracy. Other prognostic scoring models for head and neck squamous carcinoma include immune cell characteristics and compositional data [20] and integration of clinical and molecular features including pyroptosis-related genes, to design improved risk scores [21, 22]. The integration of clinical and gene expression data to improve prognostic accuracy has multiple advantages, including the combination of independent complementary predictors to improve prediction accuracy and the reduction in the number of genes needed for high-accuracy prognostic models that improve the cost-effectiveness [23]. A recent study validated a comprehensive framework for risk classification and prognosis of head and neck squamous carcinoma based on the integration of clinical and panoptosis-related genes [24]; however, reports of comprehensive prognostic scoring systems are limited. Furthermore, the integration of molecular predictors in the clinical management algorithms of HNSCC is lacking [25]. The combination of clinical and molecular features to derive high-accuracy prognostic models has been successfully applied for several cancer types including renal clear cell carcinoma, glioblastoma multiforme and bladder urothelial carcinoma, endometrial cancer, lung cancer, breast cancer and multiple myeloma are reported [26–31]. Transcriptomic signatures predicting HNSCC progression and overall survival outcomes using machine learning and related algorithms have also been reported [32–34]. However, validation is infrequently reported. Thus, developing and validating high-accuracy prognostic models for HNSCC based on clinical and molecular information is warranted. Therefore, in this study, we aimed to develop and validate a comprehensive prognostic score “Accurate Predictive Model for Overall Survival Score in Head and Neck Squamous Cancer” (APMHO) by integrating data on cancer driver gene expression and tumor phenotypic characteristics to predict the overall survival outcome. We also explored the functional pathways, mutations and immune cell landscapes associated with APMHO scores, as well as immunotherapy sensitivity markers.

## Materials and methods

### Data collection

As the training set to determine overall survival-related cancer driver genes in HNSCC, TCGA-HNSC expression data, phenotype data and overall survival data (Table 1) were downloaded using the R package TCGAmutations (v 0.3.0). The GEO dataset GSE41613 reporting on 97 HNSCC patients [23] (Table 2) was downloaded from the GEO (Gene Expression Omnibus) database (version 2.0; URL: https://www.ncbi.nlm.nih.gov/geo/). Human gene annotation data was downloaded in the gtf format from the Ensembl database (API version; URL: http://www.ensembl.org/info/data/ftp/index.html). The list of pan-cancer immune metagenes was downloaded from a previous publication [24]. The list of somatic mutations of genes in cancer was downloaded from the COSMIC (Catalogue of Somatic Mutations in Cancer) database (version 3.3; URL: https://cancer.sanger.ac.uk/census#) [25].

**Table 1:** Metadata for the TCGA-HNSCC cohort

	TCGA-HNSCC
Age (years)	
> 65	176
< = 65	325
Gender	
Male	367
Female	134
Pathologic_N	
N0	170
N1	65
N2	166
N3	7
Nx	59
NA	24
Pathologic_T	
T0	1
T1	45
T2	132
T3	96
T4	172
Tx	33
NA	22
Stage	
Stage i	25
Stage ii	69
Stage iii	78
Stage iv	261
Not reported	68
grade	
G1	62
G2	299
G3	119
G4	2
Gx	16
NA	3
TGCA-subtype	
Atypical	68
Basal	85
Classical	49
Mesenchymal	75
NA	224
HPV_Status	
Positive	36
Negative	241
NA	224
Tumor_site	
Alveolar ridge	7
Base of tongue	12
Buccal mucosa	8
Floor of mouth	25
Hard palate	5
Hypopharynx	2
Larynx	72
Lip	1
Oral cavity	49
Oral tongue	75
Oropharynx	2
Tonsil	19
NA	224
Perineural_invasion	
YES	165
NO	187
NA	149

**Table 2:** Metadata for the GSE41613 cohort

	GSE41613
Age (years)	
19–39	6
40–49	16
50–59	28
60–88	47
Gender	
Male	66
Female	31
Stage	
I/II	41
III/IV	56

### Identification of cancer driver genes

The training set was the TCGA-HNSC dataset, and the validation set was the GSE41613 dataset. Combined with Overall Survival data, expression-based ‘cancer driver’ genes significantly associated with overall survival were first screened using Cox univariate regression (coxph() function of R package survivor (v3.2-7) and ggsurvplot() function of R package survminer (v0.4.8)) with a significance threshold of p < 0.05, and associated genes were collated for subsequent analysis. Next, the significant overall survival associated cancer driver genes screened in the previous step were further validated using the GSE41613 dataset, and cancer driver genes significantly (p < 0.05) associated with overall survival in both the TCGA and GSE41613 datasets were determined as the ‘main effector’ cancer driver genes.

Identification of interacting gene pairs: Pearson correlation analysis of cancer driver gene expression values in the TCGA dataset was performed. Significantly correlated gene pairs with high correlation values were screened for subsequent analysis at a threshold of |cor|> 0.6 & adj. p < 0.05. Next, the gene pairs screened in the previous step were further validated using the GSE41613 dataset using the same threshold, and highly correlated gene pairs in both TCGA and GSE41613 were selected as the interacting gene pairs for subsequent analysis.

Combined gene-set acquisition and visualization: We obtained the union of two groups of genes (one group: the main effector cancer driver genes obtained previously; another group: interacting gene pairs). As the number of resulting genes was high, LASSO regression was utilized in this research for its ability to reduce high-dimensional transcriptomic data to a smaller subset of genes with optimized predictive capacity as an integrated prognostic model. LASSO regression was performed by using the cv.glmnet() function of R package glmnet (v4.0-2) and significantly correlated genes were obtained for subsequent modeling. The parameter setting in the cv.glmnet() function is: y = Surv(time, event), family =  ‘cox’, maxit = 20000. The main effector genes were displayed by plotting Kaplan Meier (KM) curves and gene pairs were displayed by plotting correlation scatter plots.

### Computation of ‘ACCURATE PREDICTION MODEL of AML overall survival score’ (APMHO) integrating clinical features and transcriptome score: (APMHO) and validation

Firstly, transcriptome score modeling was performed using multivariate Cox regression with a correlation threshold of p < 0.05, and transcriptome scores were calculated based on the equation 〖$Transcriptional\_Scorei= \sum_{j=1}^{n}\mathrm{expji}\times \beta j$〗, where exp denotes the expression of the corresponding gene, β denotes the regression coefficient (coef) of the corresponding gene in the multivariate Cox regression results, Transcriptional_Score denotes the expression of significantly related genes in each sample multiplied by the coef of the corresponding gene and then summed, i denotes the sample and j denotes the gene. To validate the prognostic efficacy of transcriptome scores, transcriptome scores based on TCGA samples were divided into high and low-scoring subgroups using the median as the node, and combined with overall survival data, KM curves were plotted, and p-values were calculated. A p-value < 0.05 was set as significant for the difference in overall survival probability.

Thereafter, a multivariate Cox regression analysis was performed using the Transcriptional_Score combined with age, gender, and stage of HNSCC to predict overall survival (at p < 0.05 to screen for variables significantly associated with overall survival). The Accurate Prediction Model of HNSCC was calculated based on the following formula: 〖$APMHOi= \sum_{j=1}^{n}\mathrm{factor ji}\times \beta j$〗, where factor denotes the characteristic score of the corresponding phenotype factor, β denotes the regression coefficient (coef) of the corresponding factor in the regression results, and APMHO denotes the coef of the significantly correlated factor characteristic score multiplied by the corresponding factor characteristic in each sample and then summed, i denotes the sample, and j denotes the phenotype factor. To validate the APMHO, high and low APMHO groups were identified based on the median APMHO score of the TCGA and combined with the overall survival data to plot KM curves and p-value < 0.05 was set as a significant difference in prognosis between the high and low APMHO groups. A similar analysis was performed using the APMHO scores of the GSE41613 dataset. The APMHO scores from TCGA and GSE37642 data were further used as predictions and combined with overall survival data to calculate AUC values for the model at 1, 3, and 5 years and ROC curves were plotted.

Differences in Transcriptional_Score, age, gender, grade, pathologic_N, pathologic_T, stage, Tumor_Site, Final_Hpv_Status, TGCA-subtype and perineural_incasion in high and low APMHO score groups were determined using Kruskal Wallis test and Fisher test and heat maps were drawn. In addition, the APMHO score differences associated with age, gender, grade, pathologic_N, pathologic_T, stage, Tumor_Site, Final_Hpv_Status, TGCA-subtype, perineural_incasion and Transcriptional_Score groupings were determined using Wilcoxon's test and Kruskall Wallis test, and box plots were drawn.

To validate the APMHO, Transcriptional_Score, age, gender, and stage were subjected to Univariate Cox regression analysis and significant factors were identified. Next, multivariate Cox regression analysis was applied to further demonstrate that the significant variables within the APMHO score were independent prognostic variables. The results were depicted as column line plots drawn using the R packages rms (v 6.1-0), survival (v 3.5-5) and regplot (v 1.1), first constructing the Cox proportional risk regression model with cph(), then calculating the overall survival probability with the survivor() function, and finally constructing the column line plot object with the regplot() function to plot a correction curve. Decision curve analysis (Decision Curve Analysis (DCA)) was performed with the decision_curve() function in the R package rmda (v 1.6) to plot the decision curve.

### Potential molecular mechanisms linked to APMHO scores: mutational landscape and enriched functional pathways

Mutational landscapes linked to high and low APMHO score grouping were determined. MAF files for TCGA-HNSCC mutations were combined with high and low APMHO groupings to map the mutation landscapes using the oncoplot () function of R package maftools (v3.17). Genes with mutations in at least 1 sample were obtained, differences in mutations between high and low APMHO groups were calculated, and the significance of differences was tested using the Fisher test. To determine hallmark functional pathways linked to APMHO grouping, ssGSEA was performed using Hallmark gene sets (MSigDB v7.5.1), and the enrichment scores were obtained. Heatmaps were plotted for display and the significance of differences was tested using Wilcoxon's test.

### Tumor-infiltrating immune cells associated with APMHO scoring

The enrichment scores of 28 immune infiltrating cells in cancer samples were calculated using the R package GSVA (v 3.17) based on ssGSEA, and data were normalized using the scale() function. Data for high and low APMHO groups were displayed using box plots. In addition, the Pearson correlation coefficient between APMHO scores and immune infiltrate enrichment scores was calculated and bubble plots were drawn.

### Tumor immune evasion and cancer immunotherapy response associated with AMPAO scores

To assess the possible value of APMHO scores in predicting sensitivity to cancer immunotherapy, expression of immune checkpoint genes, interferon-gamma pathway markers, m6A regulators and correlation with Tumor Immune Dysfunction and Exclusion (TIDE) dysfunction scores [26] were analyzed [27]. Interferon-gamma pathway markers and m6A regulators were collected from the literature and differences in the expression of genes associated with high and low APMHO groups in the TCGA-HNSCC data were analyzed using Wilcoxons's test. The TIDE web resource (http://tide.dfci.harvard.edu) was used to calculate the TIDE scores for the TCGA samples and determine the correlation with the APMHO scores and was depicted by scatter plots. Additionally, to determine potential differences in immunotherapy responses, the IMvigor210 bladder cancer data were downloaded using the R package IMvigor210CoreBiologies (v 1.0.0). Transcriptome scores were calculated based on genes present in the transcriptome score model. With the score median as the node, samples were grouped into high and low-score subgroups. KM curves and ROC curves were plotted. Differences in transcriptome scores between the response subgroups were assessed and plot box plots were plotted. Immunophenoscore analysis (IPS) [35] predicts the overall immunogenicity and tumor response to checkpoint inhibitors, where a higher score represents better tumor response. IPS analysis was performed by data retrieval from The Cancer Immunome Atlas (TCIA, https://tcia.at/home).

## Results

### Identification of cancer driver genes

**Fig. 1:**
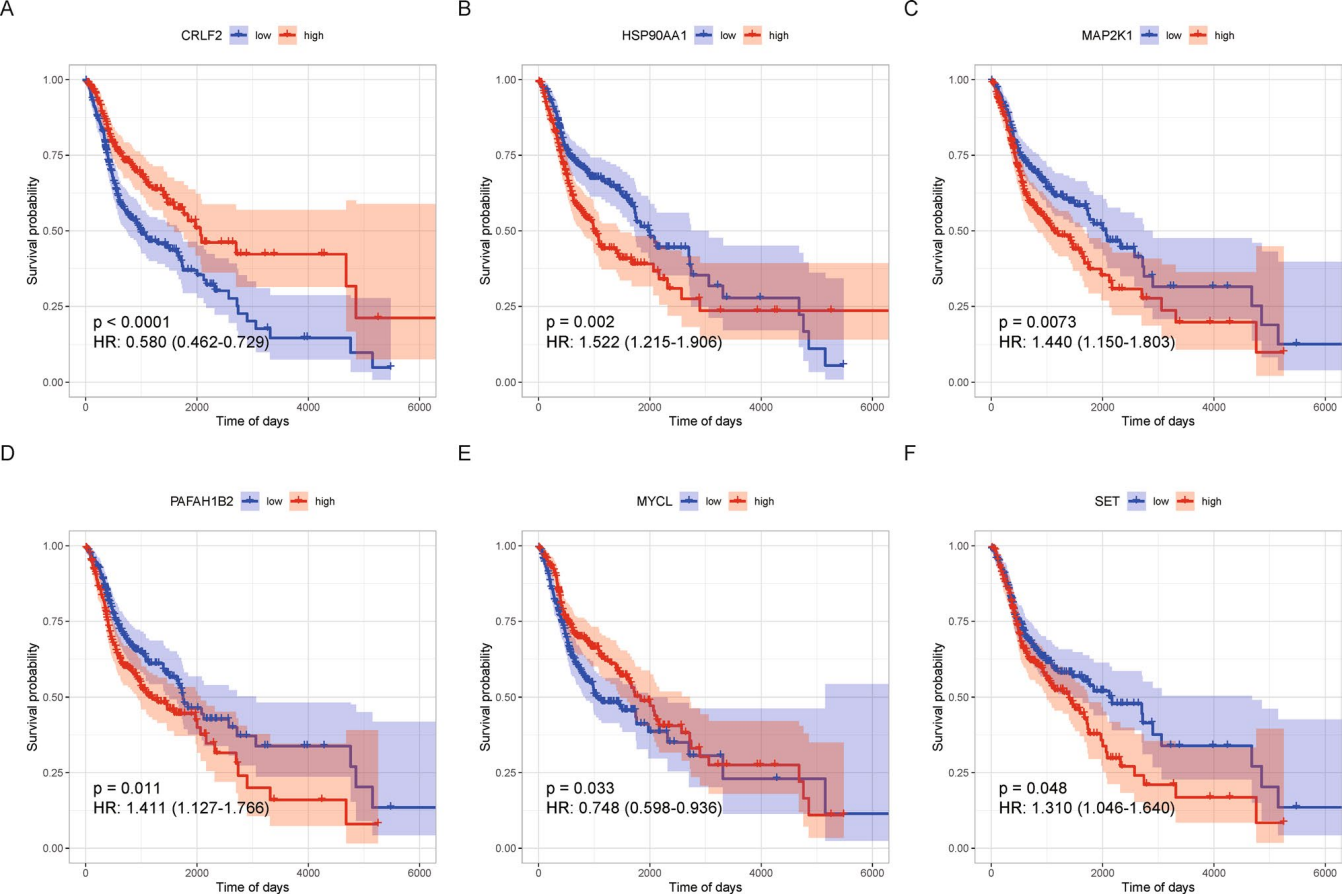
Kaplan Meiyer curves for 6 main effector genes (i.e., CRLF2 (**A**), HSP90AA1 (**B**), MAP2K1 (**C**), PAFAH1B2 (**D**), MYCL (**E**), SET (**F**)) selected using LASSO regression analysis

**Fig. 2:**
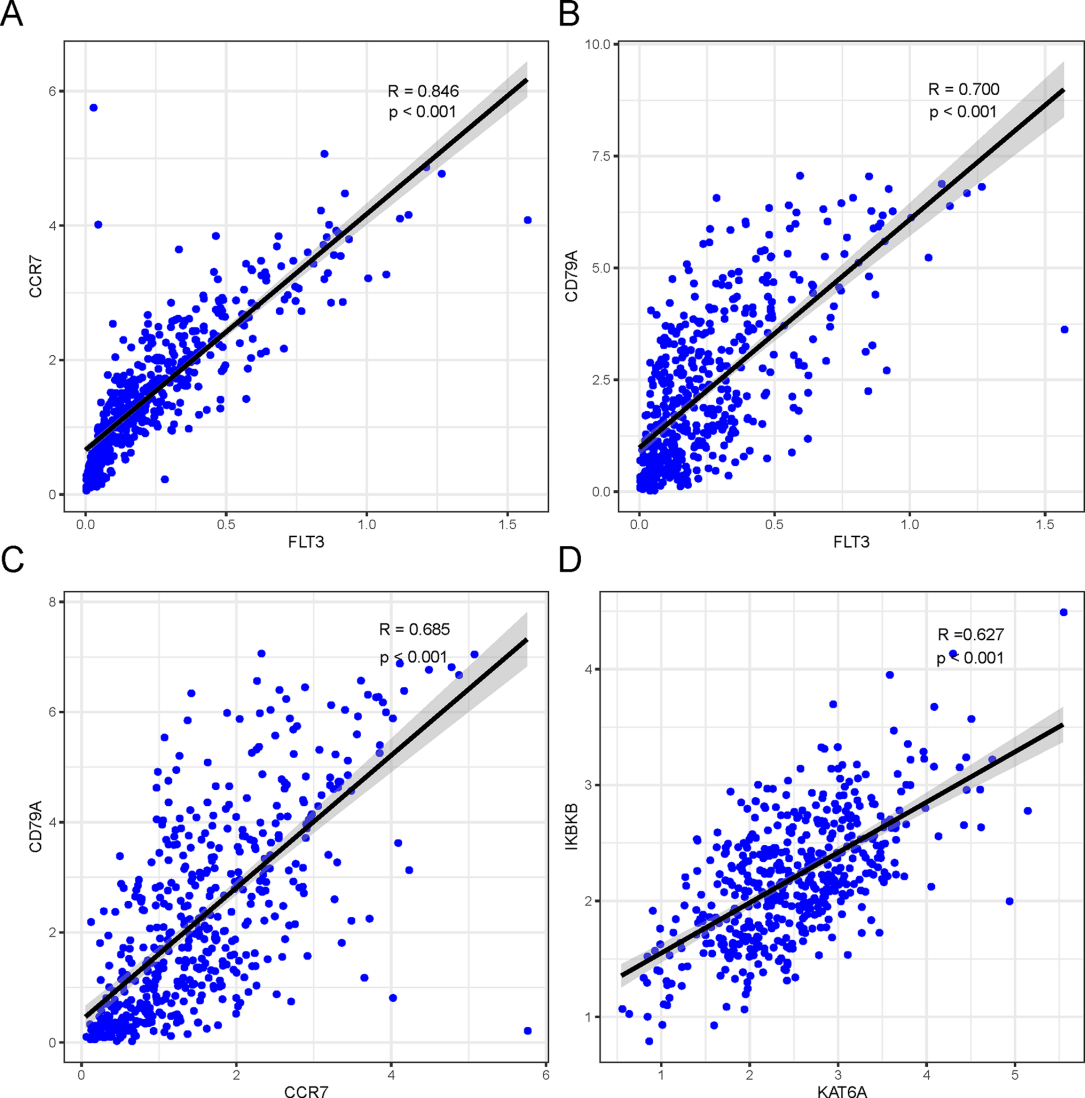
Strongly interacting gene pairs selected (i.e., CCR7 & FLT3 (**A**), CD79A & FLT3 (**B**), CD79A & CCR7 (**C**), and IKBKB & KAT6A (**D**)) using LASSO regression analysis

Relevant TCGA-LAML expression data and clinical data were downloaded from the UCSC Xena database (version 1.0; URL: http://xena.ucsc.edu/), yielding data for 501 cancers. The COSMIC database provided 733 genes in total, of which 711 had expression information in TCGA. A univariate Cox regression analysis of the 711 genes yielded 107 genes significantly associated with overall survival, which were used in subsequent analyses (Supplementary Table 1). These 107 primarily screened genes were further validated in the GSE41613 dataset to obtain 20 genes that were significantly associated with overall survival in both datasets as main effectors (Supplementary Table 2 shows the validation results in GSE41613). Interacting gene pairs among the cancer driver genes were determined in the TCGA dataset at a correlation threshold (|cor|> 0.6 & adj. p < 0.05) to obtain 4516 pairs of gene pairs with strong reciprocal relationships for subsequent analysis (Supplementary Table 3). Further validation of these 4516 gene pairs in the GSE41613 dataset yielded 402 gene pairs with strong interactions in both datasets, containing 129 genes in total (Supplementary Table 4). The selected main effector and interacting genes were merged to obtain 144 final genes. Downscaling was performed with LASSO regression to obtain 11 genes, where CRLF2, HSP90AA1, PAFAH1B2, MYCL and DAXX were the main effector genes. CCR7-FLT3, CD79A-FLT3, CD79A-CCR7 and IKBJB-KAT6A were interacting genes and had strong reciprocal interactions. (Figs. 1, 2, Supplementary Fig. 1).

### Computation of ‘accurate prediction model of HNSCC overall survival score’ (APMHO) integrating clinical features and transcriptome score: (APMHO) and validation

**Fig. 3:**
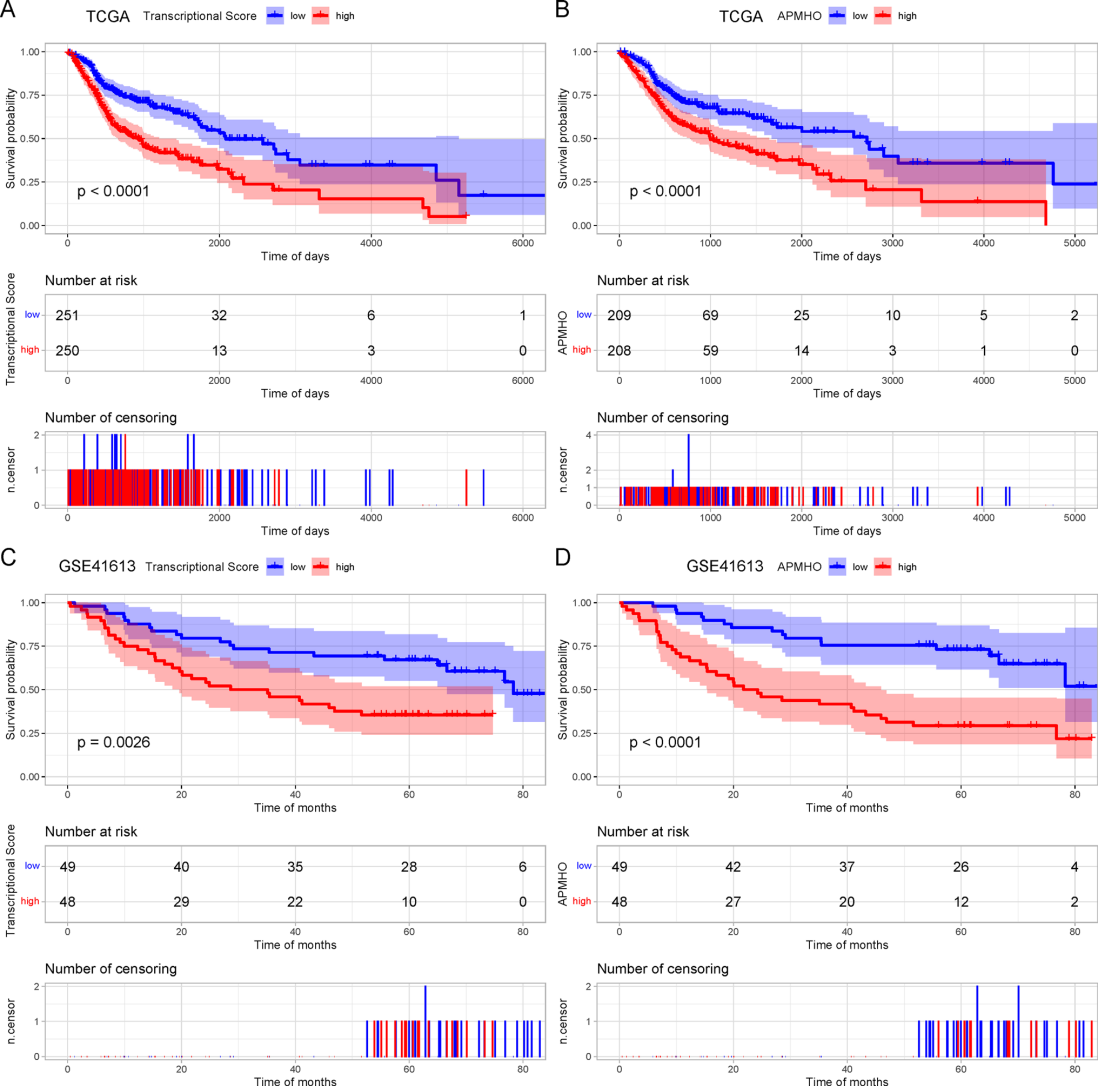
Kaplan Meiyer curves for transcriptional score grouping and high and low APMHO grouping for TCGA data (**A**, **B**) and GSE41613 data (**C**, **D**)

Multivariate Cox regression analysis performed on 11 genes obtained 3 significant genes at p < 0.05. Modeling with their correlation coefficients provided Transcriptional_Score = 0.5402*MAP2K1 + (− 0.1759) * MYCL + 0.3264* PAFAH1B2. Grouping based on the median Transcriptional_Score showed KM curves differed significantly for the high and low groups (Fig. 3A).

**Fig. 4:**
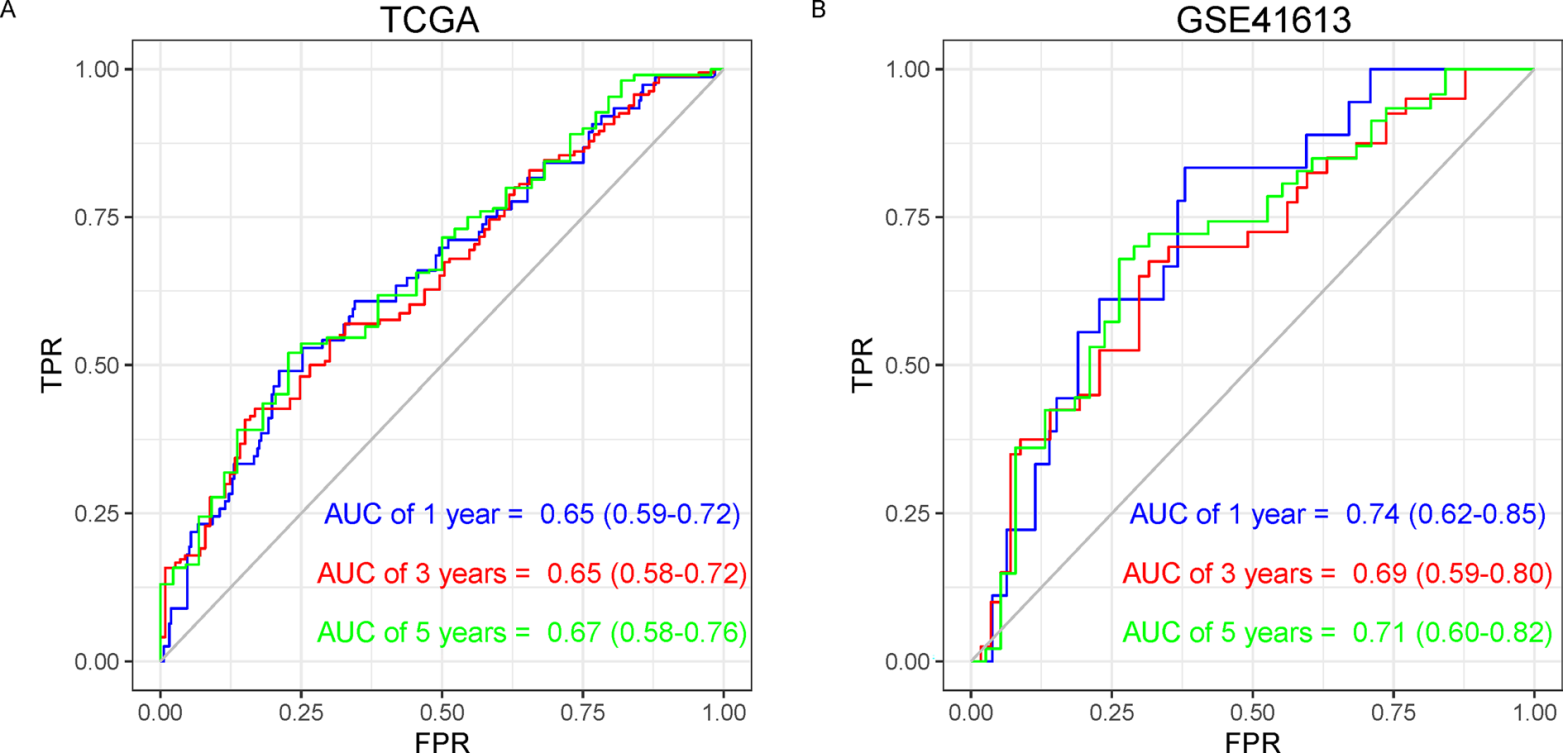
ROC curves of APMHO scores for TCGA data (**A**) and GSE41613 data (**B**)

Further combining Transcriptional_Score, age, gender, and stage for multivariate Cox regression analysis, Transcriptional_Score, age and stage were identified as significant factors, so the final APMHO score was constructed using these 3 elements, i.e. APMHO = 0.8904 * Transcriptional_Score + 0.0224 *age + 0.7614*stage. The median APMHO score value was used to divide the samples into the high and low-risk groups, and the KM curves of the high and low groups differed significantly (Fig. 3B). The AUCs of the model at 1, 3 and 5 years were all greater than 0.65 (TCGA_HNSCC data: AUC_1 year = 0.65; ACU_3 year = 0.65; AUC_5 year = 0.67; GSE41613 dataset: AUC_1 year = 0.74; ACU_3 year = 0.69; AUC_5 year = 0.71), indicating general model efficacy (Fig. 4A).

**Fig. 5:**
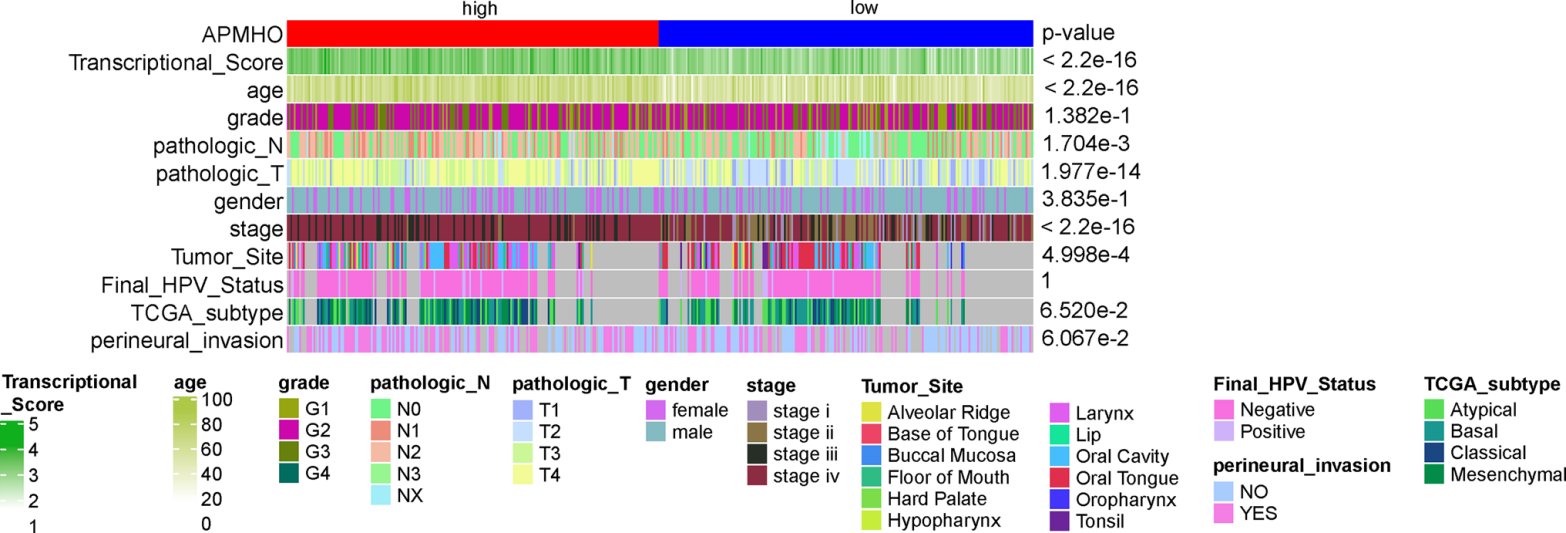
Differences between high and low APMHO groups in phenotypical variables

**Fig. 6:**
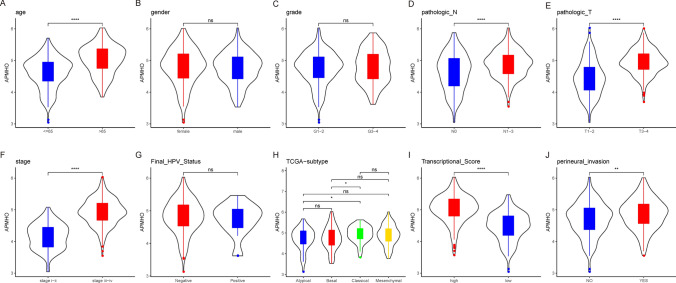
Differences in APMHO scores between subgroups by phenotype (i.e., age (**A**), gender (**B**), grade (**C**), pathologic_N (**D**), pathologic_T (**E**), stage (**F**), final_HPV_status (**G**), TCGA_subtype (**H**), transcriptional_score (**I**), and perineural_invasion (**J**)). **** indicates p < 0.0001; *** indicates p < 0.001; ** indicates p < 0.01; * indicates p < 0.05

In the TCGA data, high and low APMHO subgroups showed significant differences in Transcriptional_Score, age, gender, grade, pathologic N, pathologic T, stage, Final HPV status, TCGA-subtype and perineural invasion (Fig. 5). The mean APMHO scores were significantly different between Transcriptional Score, age, gender, pathologic N, pathologic T, stage, Tumor Site status subgroups (Fig. 6).

**Fig. 7:**
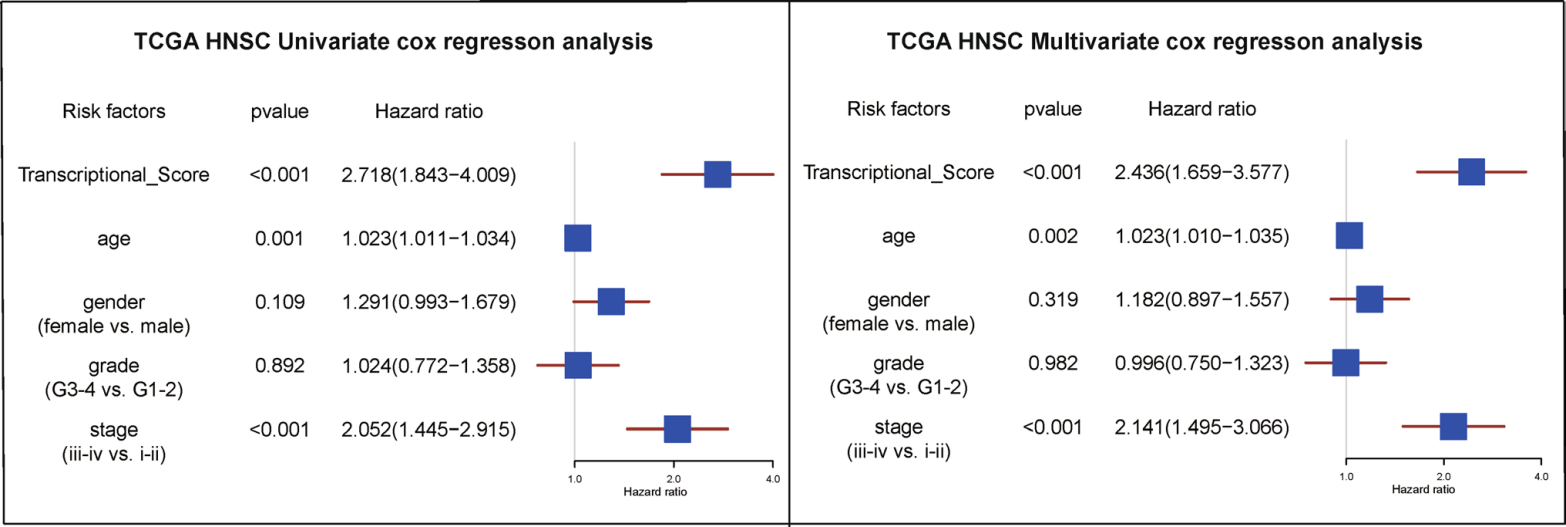
Forest plots for Cox regression analysis using AMPAO components as predictors of OS

**Fig. 8:**
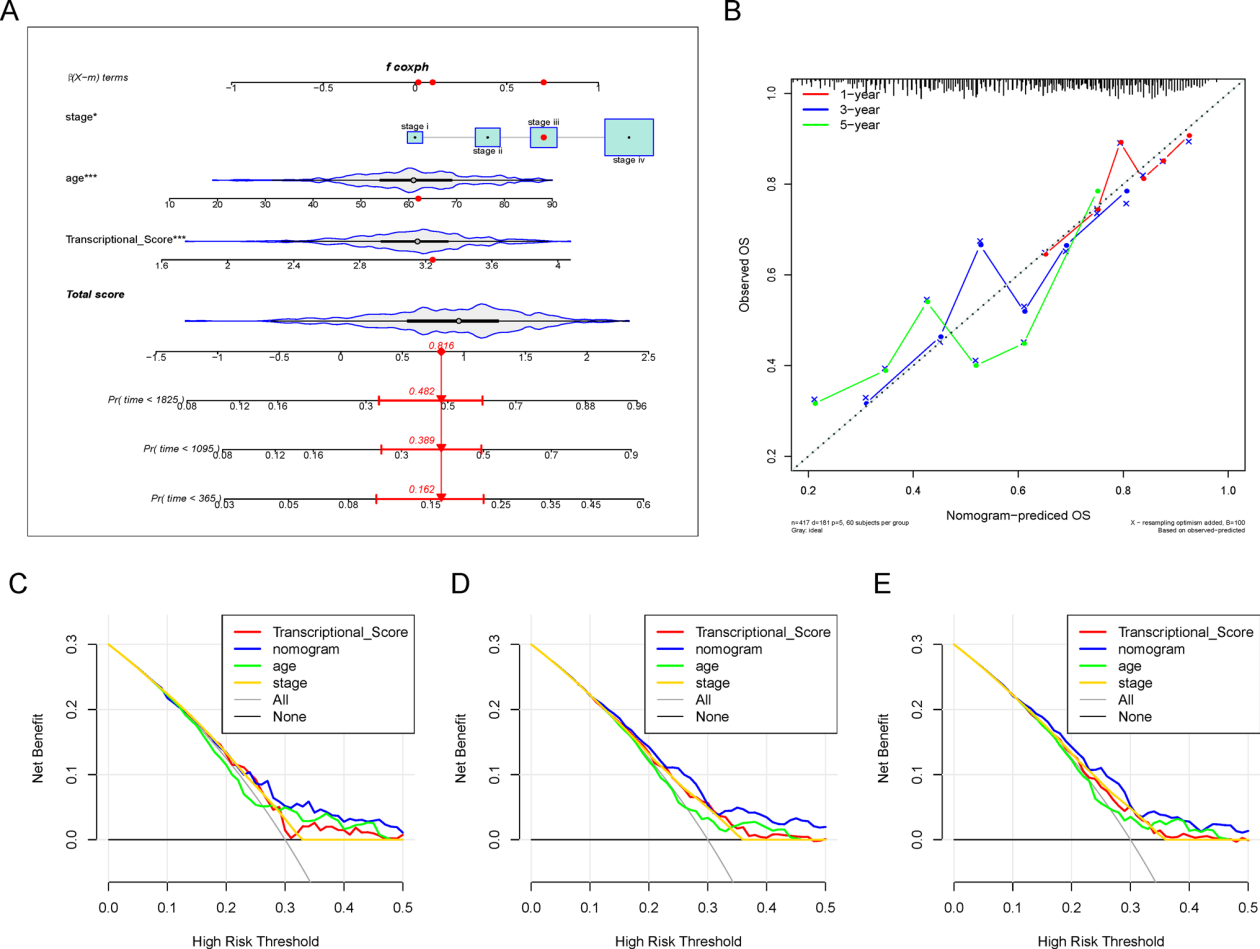
Decision curve analysis: **A** column line plot based on the multivariate Cox regression model; **B** calibration curves for 1-, 3-, and 5-year OS; **C**–**E** decision curves for 1, 3, and 5 year OS

Cox regression analysis showed that Transcriptional Score, age, gender, grade, and stage were independent prognostic factors, consistent with the results for the APMHO scores (Fig. 7). Further, decision curve analysis showed a higher net benefit of Transcriptional Score, age, and stage over other variables, indicating APMHO had the best decision efficacy and served as the main influencing factor (Fig. 8).

### Potential molecular mechanisms linked to APMHO scores: mutational landscape and enriched functional pathways

**Fig. 9:**
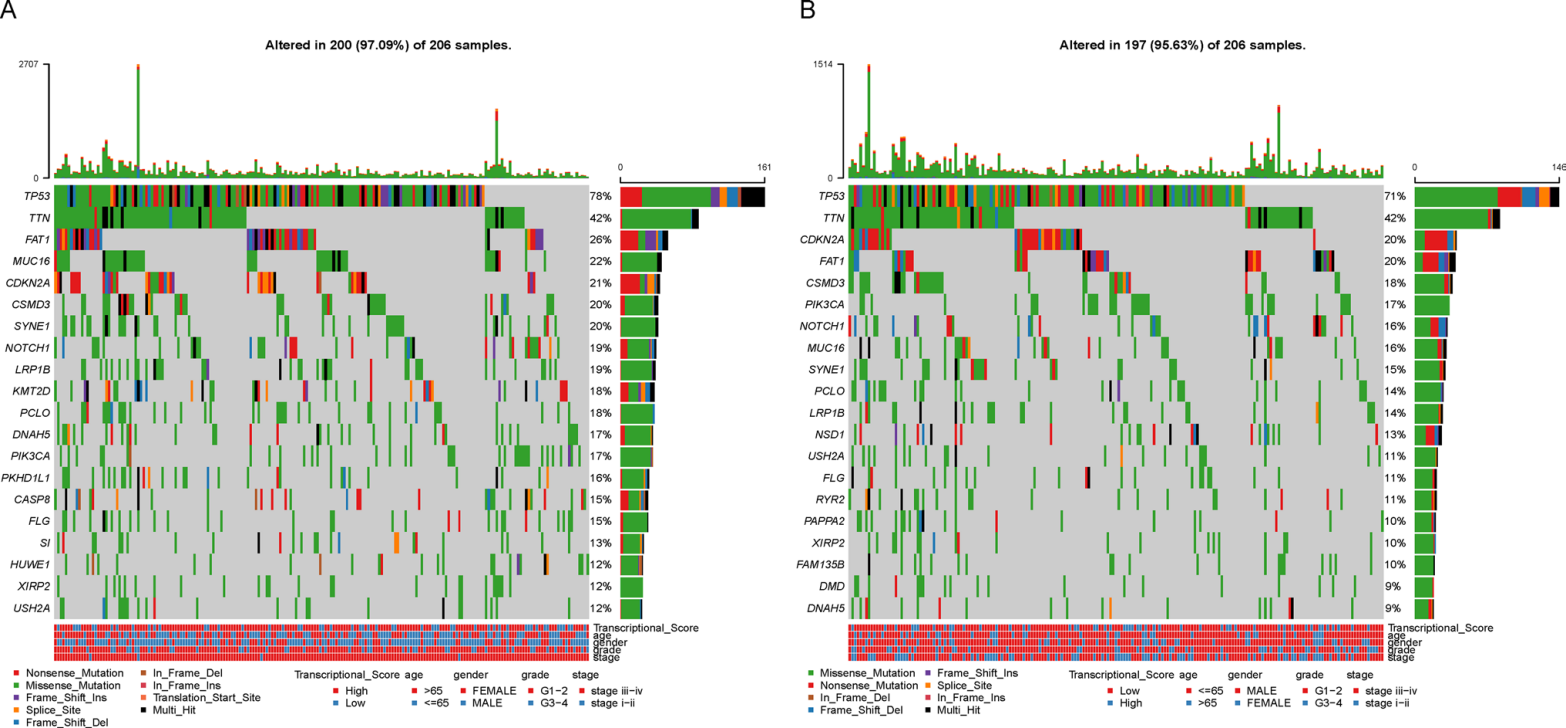
Mutational landscape of the high and low APMHO groups. **A** High APMHO group; **B** Low APMHO group

Mapping the mutational landscape of TCGA samples to high and low APMHO grouping showed that the mutation rate of samples in the high APMHO group was higher than that in the low APMHO group. The TP53 gene showed the highest mutation rate, seen in the high APMHO group (78%) and low APMHO group (71%), followed by TTN, which also had a high mutation rate in the cancer group (14%). The differences in gene mutation rates between the high and low APMHO groups are shown in Supplementary Table 5 and Fig. 9.

### Tumor-infiltrating immune cells associated with APMHO scoring

**Fig. 10:**
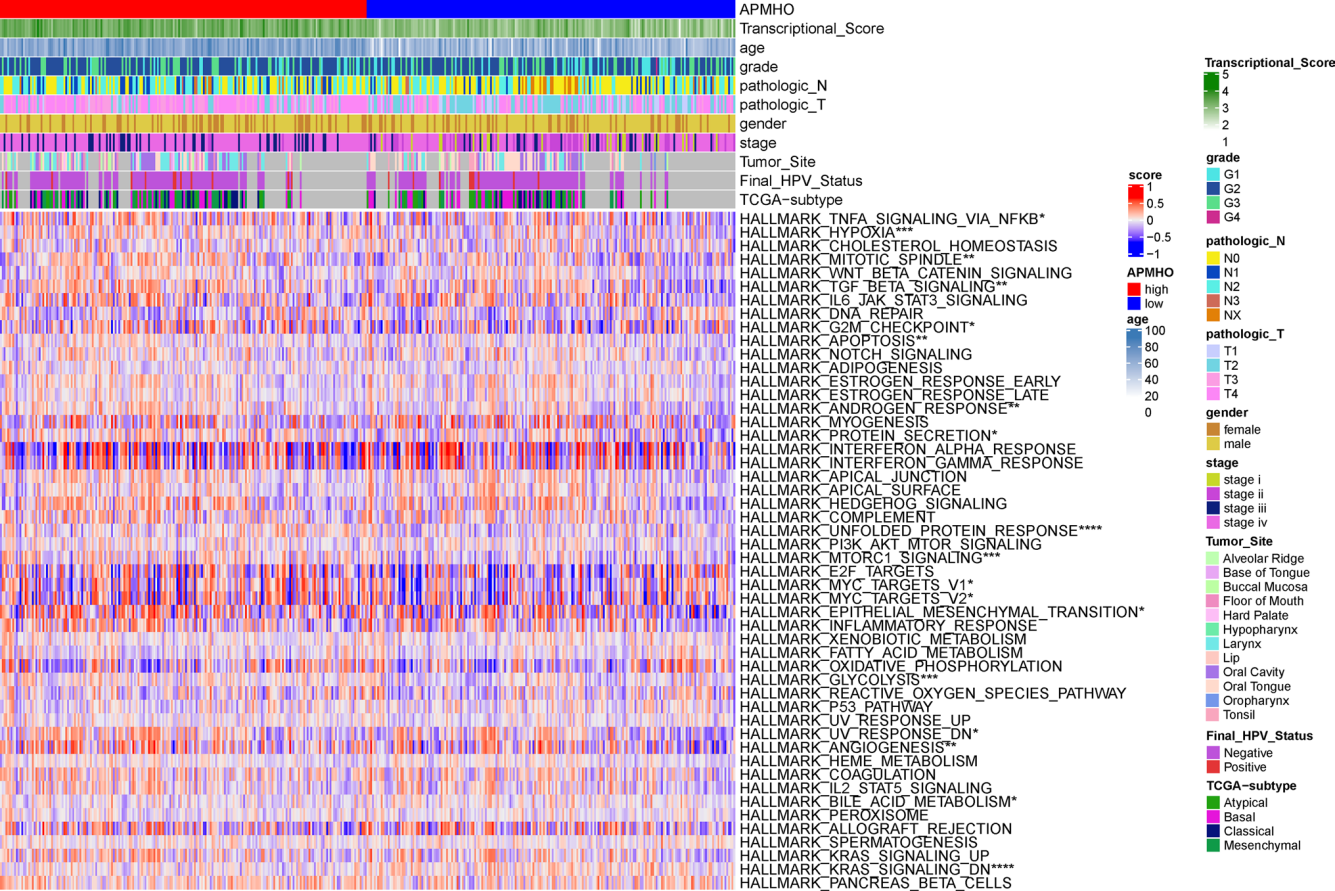
Heat map showing differences between high and low APMHO groups. for hallmark pathways enrichment. **** indicates p < 0.0001; *** indicates p < 0.001; ** indicates p < 0.01; * indicates p < 0.05

Enrichment scores were calculated for 28 immune infiltrating cells and the results showed significant differences between the high and low APMHO groups for 28 immune infiltrating cells. Activated CD4 T cells and immature dendritic cells had higher enrichment scores in the high-APMHO group than in the low-APMHO group (Fig. 10).

**Fig. 11:**
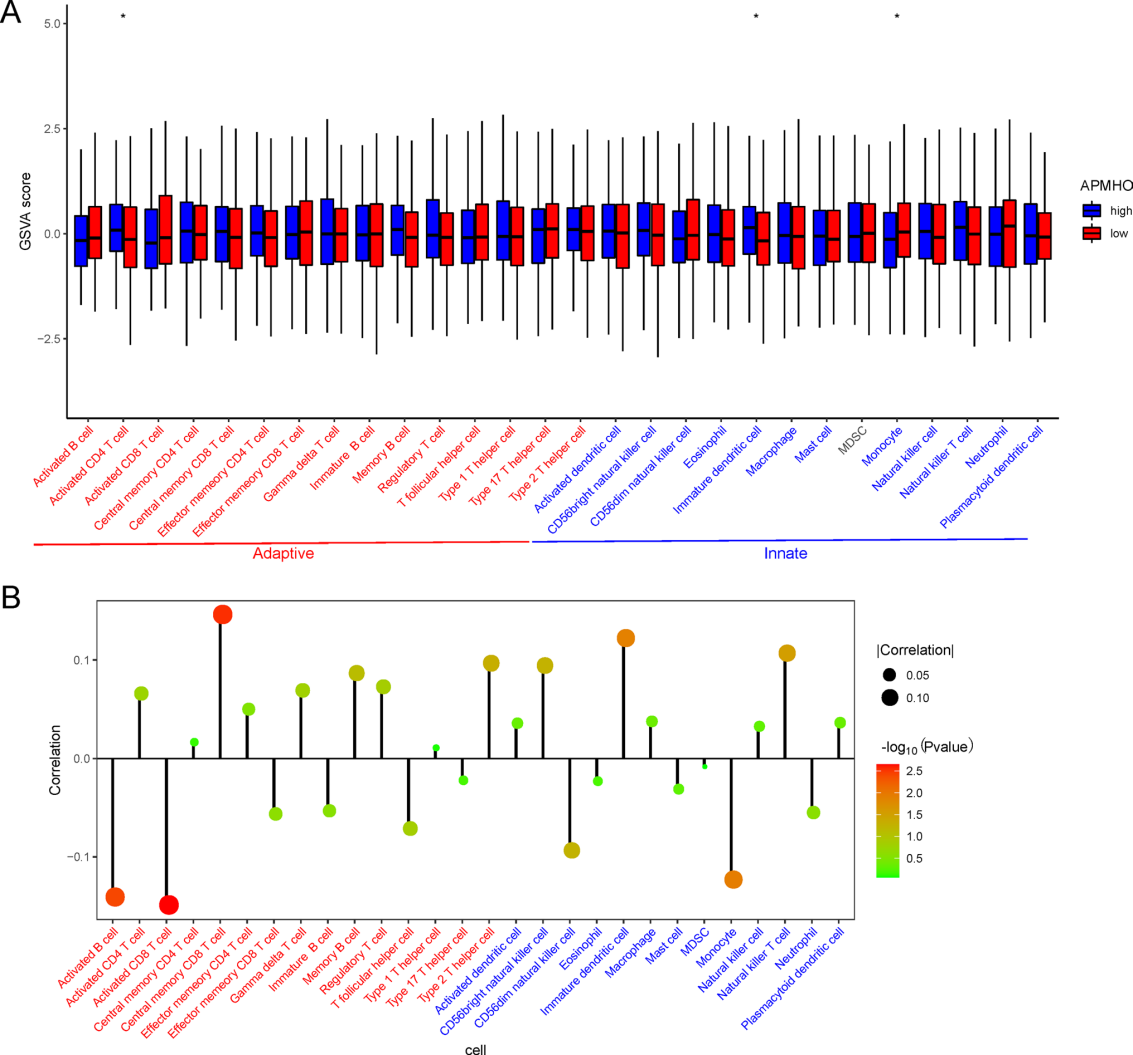
Immune infiltrating cells associated with APMHO scores. **A** Differences in immune infiltrating cell enrichment score in high and low APMHO groups; **B** Correlation between APMHO scores and immune infiltrating cell scores

In addition, the correlation between the APMHO score and immune infiltrating cell enrichment score showed that the APMHO score was significantly negatively correlated with Activated B cells, Activated CD8 T cells; however, it was significantly positively correlated with Central memory CD8 T cell, Immature dendritic cell and Natural killer T cell (Fig. 11).

### Tumor immune evasion and cancer immunotherapy response associated with AMPAO scores

**Fig. 12:**
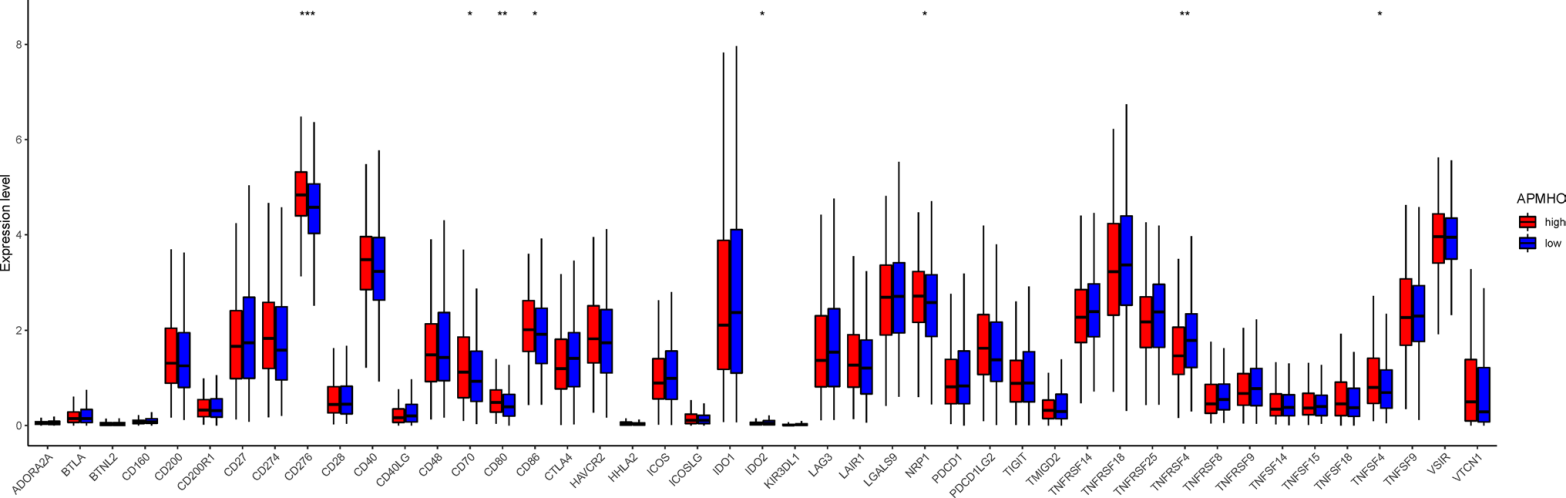
Differences in immune checkpoint expression between high and low APMHO groups. **** indicates p < 0.0001; *** indicates p < 0.001; ** indicates p < 0.01; * indicates p < 0.05

Immune checkpoint gene analysis showed that 8 out of 45 immune checkpoints were differentially expressed between APMHO subgroups. CD276, CD70, CD86, NPR1 and TNFSF4 had significantly higher expression in the high APMHO subgroup, while IDO2 and TNFRSF4 had significantly higher expression in the low APMHO subgroup (Fig. 12).

**Fig. 13:**
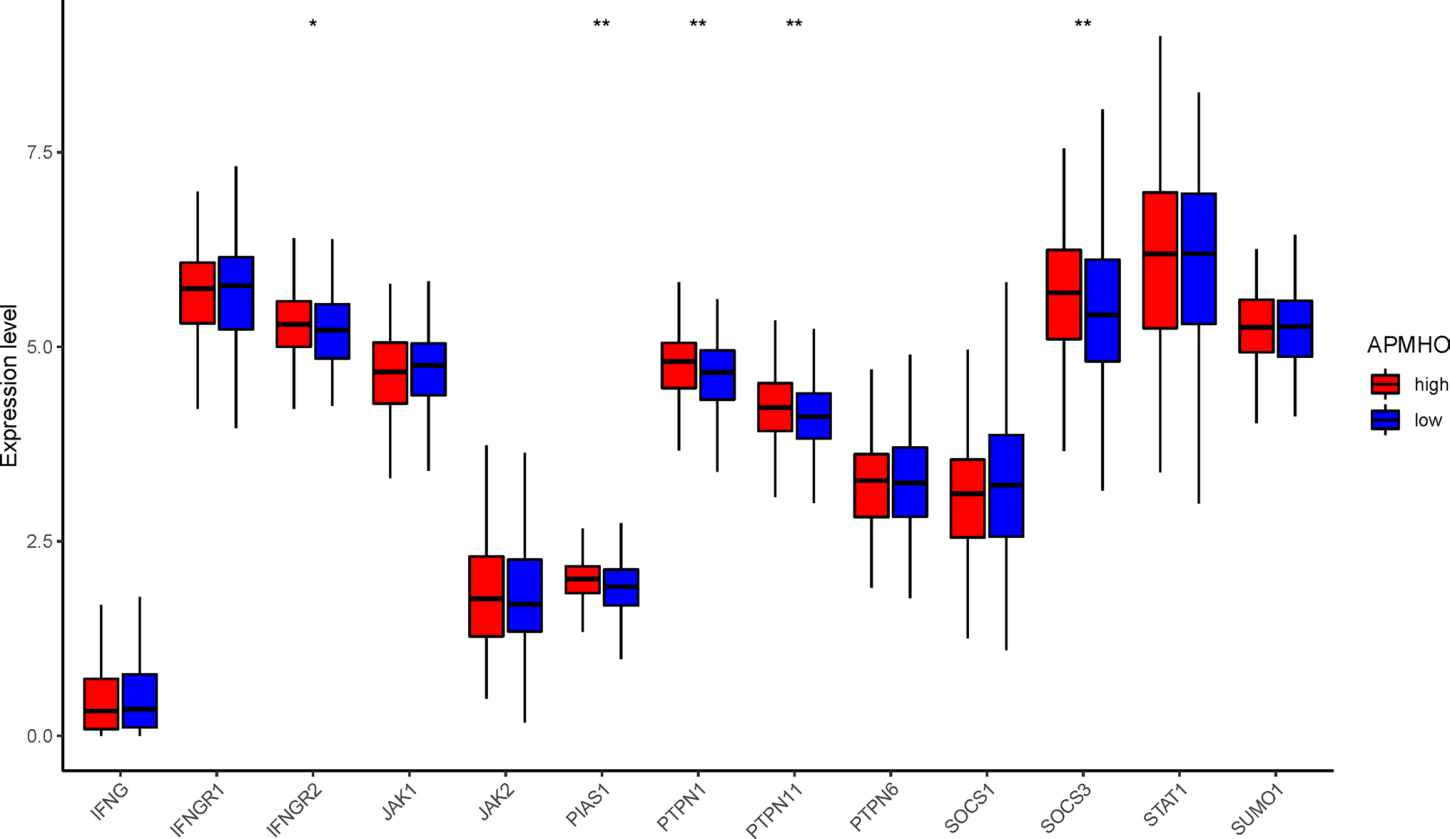
Differences in interferon-γ pathway marker expression between high and low APMHO groups. **** indicates p < 0.0001; *** indicates p < 0.001; ** indicates p < 0.01; * indicates p < 0.05

**Fig. 14:**
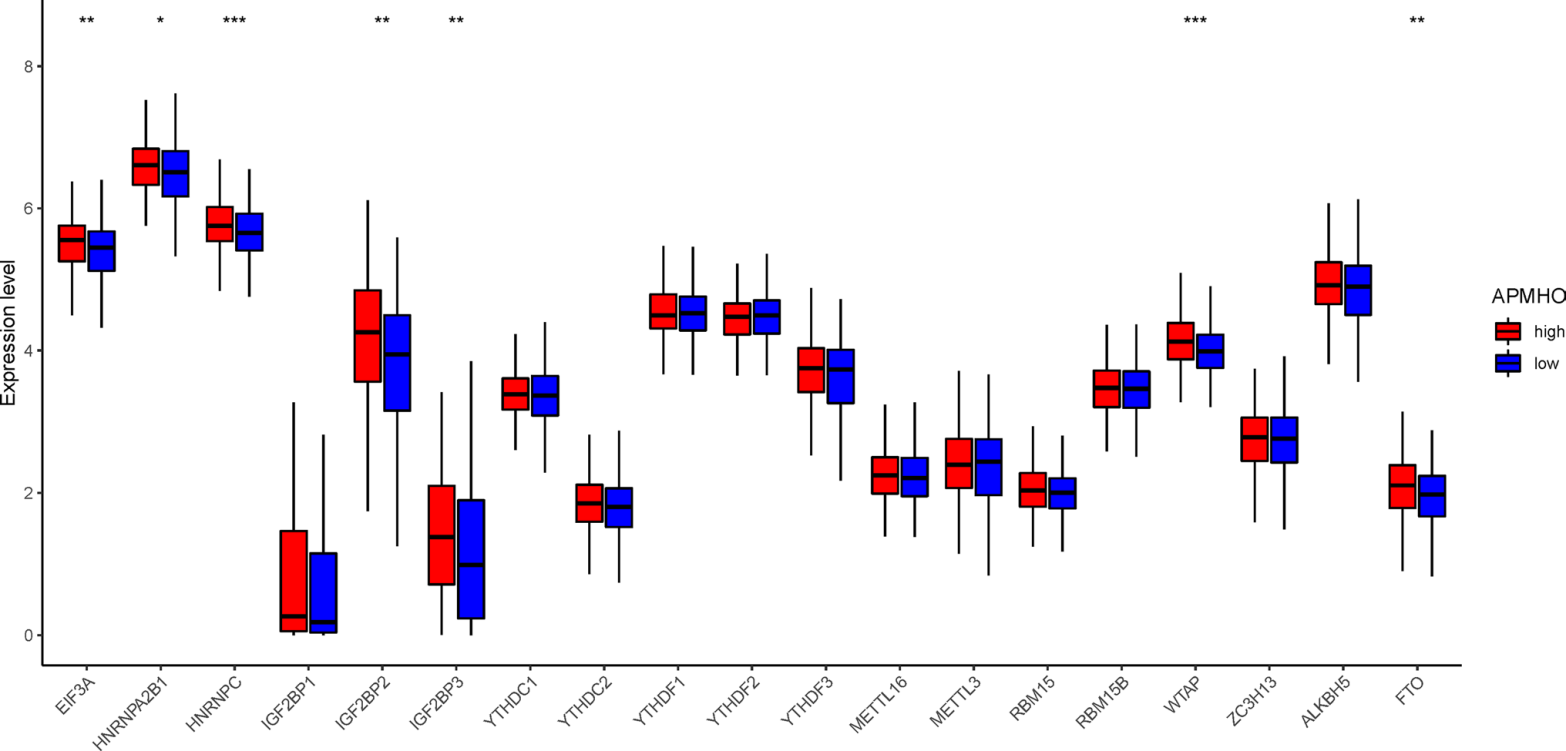
Differences in m6A regulator expression between high and low APMHO groups. **** indicates p < 0.0001; *** indicates p < 0.001; ** indicates p < 0.01; * indicates p < 0.05

The analysis of interferon-γ pathway markers showed that 5 of the 13 interferon-γ pathway markers were differentially expressed, with IFNGR2, PTPN1, PTPN11, and SOCS3 showing significantly higher expression in the high APMHO group. The correlation results indicated a significant negative correlation between APMHO and Activated CD8 T cells (Fig. 13). Similarly, 7 out of 19 m6A regulators were differentially expressed between APMHO groups, among which EIF3A, FTO, HNRNPA2B1, HNRNPC, IGF2BP2, IGF2BP3, and WTAP had significantly higher expression in high APMHO (Fig. 14).

### Correlation of IPS score differences with APMHO scoring

**Fig. 15:**
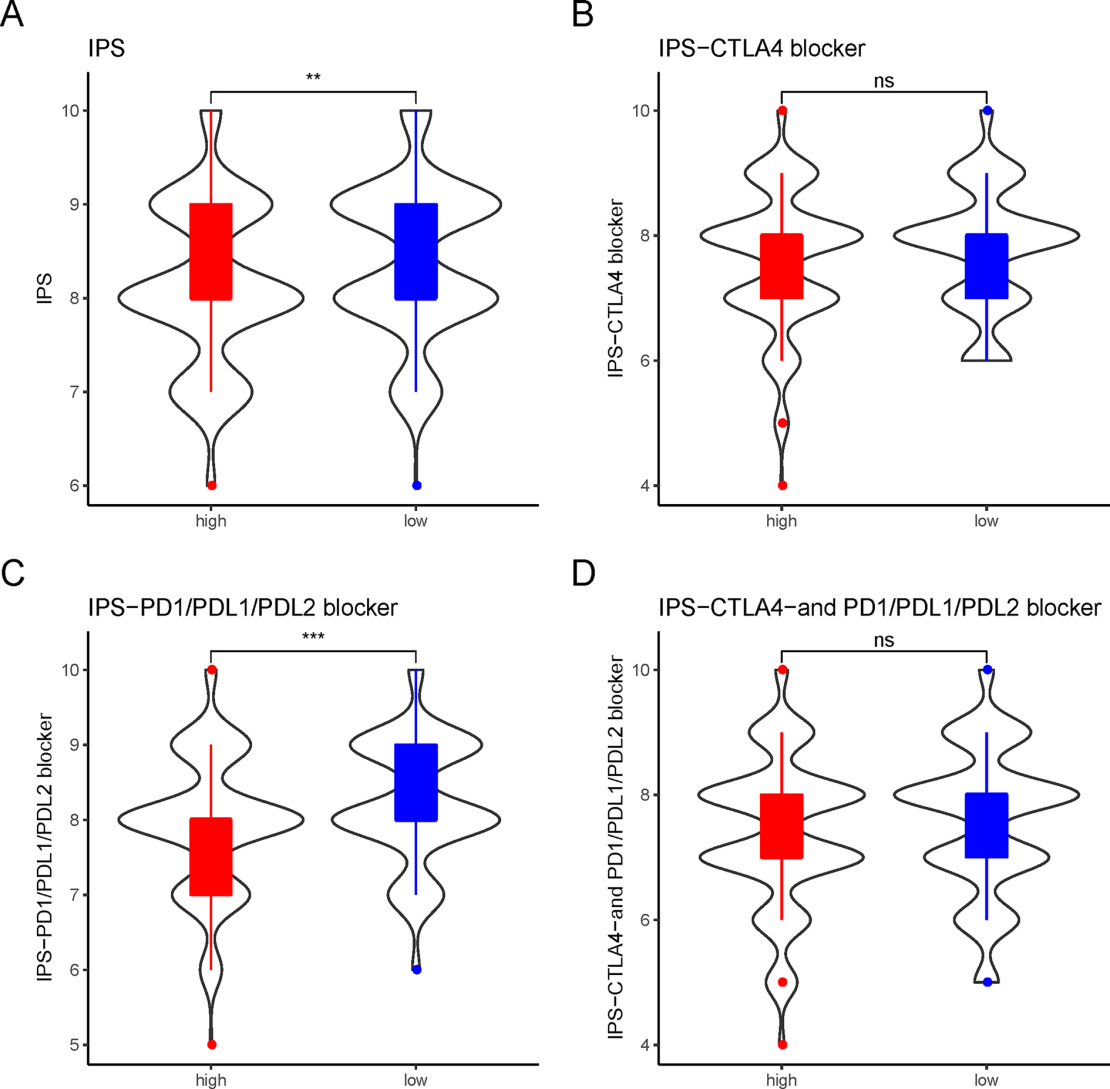
Differences in IPS scores (i.e., IPS (**A**), IPS-CTLA4 blocker (**B**), IPS-PD1/PDL1/PDL2 blocker (**C**), IPS-CTLA4- and PD1/PDL1/PDL2 blocker (**D**)) between high and low APMHO groups. *** indicates p < 0.001; ** indicates p < 0.01

Further examination of the differences in IPS scores between the high and low APMHO groups showed that the low APMHO samples had significantly higher IPS scores than the high APMHO group; the low APMHO samples had significantly higher IPS-PD1/PDL1PDL2 blocker scores than the high APMHO group; while IPS-CTLA4 blocker and IPS-CTLA4- and PD1/PDI.1/PDL.2 blocker did not differ significantly between the high and low APMHO groups. The higher IPS implied that the relevant samples were more effective for immunotherapy, and the results implied that the samples in the low APMHO group were more effective for immunotherapy, especially for PD1/PDL1/PDL2 inhibitor-based immunotherapy. (Fig. 15).

## Discussion

This study developed a transcriptomic-clinical prognostic model termed APMHO for predicting overall survival in head and neck squamous cell carcinoma. By integrating expression of six cancer driver genes with patient age and tumor stage, the APMHO score demonstrated good discrimination between high and low risk groups in both discovery and validation cohorts. The model provides a robust personalized prognostication approach by incorporating complementary clinical and molecular data. Associations with tumor biological differences further supported its relevance. With additional independent validation, the APMHO score could enable improved individualized risk stratification to guide management and precision medicine for HNSCC patients.

Existing prognostic models for head and neck squamous cell carcinoma (HNSCC) have several limitations and gaps that undermine their accuracy and clinical applicability. These include the lack of comprehensive assessment, limited consideration of heterogeneous patient populations, absence of external validation and applicability, inconsistent endpoints and follow-up durations, failure to consider treatment factors and the tumor microenvironment, and lack of usability and accessibility. One notable and significant limitation is the lack of comprehensive assessment. Many models focus on limited variables such as tumor stage and clinical factors, which may not capture the full complexity of HNSCC. Important prognostic factors such as molecular markers, genetic abnormalities, or biomarkers are often overlooked, leading to incomplete prognostic assessments. This limited assessment may result in inaccurate predictions of patient outcomes and hinder the development of personalized treatment strategies. Addressing these limitations and gaps in existing prognostic models for HNSCC is of paramount importance, and will enable more personalized treatment strategies, better prognostic predictions, and improved patient outcomes in the management of HNSCC. The present study described and validated a comprehensive score APMHO for HNSCC prognosis based on cancer driver genes, phenotype and clinical information. The two major strengths of the score were the incorporation of clinical and transcriptomic features, which form independent and complementary datasets, and the improved decision performance of the comprehensive score, as compared to the transcriptomic score alone. The score computation was done utilizing a multivariate Cox regression approach that has been the most widely accepted tool for prognosis and survival prediction in oncology [36–38]. The prognostic model developed in the present research leverages both molecular gene expression data as well as independent clinical predictors to achieve improved accuracy for predicting overall survival outcomes. By combining cancer gene expression patterns with age and stage, the APMHO score showed good discrimination ability between high and low risk groups in both the training and validation cohorts. The model demonstrated the utility of a personalized, precision medicine approach by linking transcriptomic variability with differences in clinicopathological and biological features. The present study provides a strong basis for further advancing integrated omics-clinical prognostic models to guide individualized management in head and neck cancer.

This study found associations between the APMHO prognostic score and immune features in head and neck squamous cell carcinoma that provide clues into potential underlying mechanisms relating tumor immunity to disease outcomes. The observed differences in immune checkpoint proteins like PDL1 and CTLA4 indicate greater impedance of anti-tumor immunity in the high AMPAO group, consistent with prior studies linking PDL1 expression to poor prognosis and immunotherapy response in HNSCC. Meanwhile, heightened interferon signaling and mutations in genes like TTN have been tied to resistance to checkpoint inhibitors [55]. Collectively, the immune profiles associated with high AMPAO scores suggest an exhausted T-cell phenotype and highly immunosuppressive tumor microenvironment, which aligns with worse immunotherapy response as observed through lower IPS scores, providing insight into precision immunotherapy implications. The high APMHO group showed possible greater immune evasion evidenced by higher expression of inhibitory immune checkpoints like CD276 and activation of immunosuppressive pathways like mTORC1. This group also showed relative enrichment in suppressive immune cell types including immature dendritic cells. In contrast, the low risk group had features indicating enhanced immune activity like higher CD8 T cell activation. The lower IPS (immunophenoscore) in the high-risk APMHO group further suggests reduced immunogenicity and immunotherapy sensitivity. Together, these findings indicate the APMHO score may integrate the transcriptomic impact of tumor-immune interactions influencing survival.

At the outset, LASSO regression identified 11 genes of significant predictive value. Thereafter, a 6-gene signature of AML prognosis was identified, comprising CRLF2, HSP90AA1, MAP2K1, PAFAH1B2, MYCL and SET genes, and using multivariate Cox regression analysis, three genes, MAP2K1, MYCL and PAFAH1B2 were selected for the Transcriptional Score computation. The involvement of most of these genes as cancer drivers in HNSCC is supported by previous literature. DAXX gene is implicated in malignant transformation and tumor promotion [39]. The release of FMS-like tyrosine kinase 3 ligand or FLT3L by natural killer cells is found to enhance HNSCC response to radioimmunotherapy [40]. Its related protein, CC chemokine receptor 7 (CCR7), has been associated with worse survival in HNSCC [41]. The B cell receptor CD79A is implicated in tumor promotion by myeloid cells [42]. The oncogene KAT6A promotes cancer by inhibiting cellular senescence [43]. Among the 6 signature genes, CRLF2 as part of an immune score-related risk signature, performed well in the prognostic stratification of OSCC patients and could effectively distinguish their survival status [44]. HSP90AA1 was highly expressed in various tumors and significantly correlated with poor prognosis [45]. HSP90AA1/IL-17 signaling pathway was associated with cisplatin resistance in head and neck squamous carcinoma [46]. MAP2K1 activation is implicated in several malignancies and is a therapeutic target in HNSCC [47]. PAFAH1B2 as one of the catalytic subunits of type I PAF-AH (Platelet-activating factor acetylhydrolase) was associated with poor prognosis and affected proliferation in hypopharyngeal squamous cell carcinoma [48]. MYCL is a member of the MYC proto-oncogene family and regulates cellular programs orchestrating multiple hallmarks of cancer, including proliferation, metabolism, invasiveness, and immune surveillance [49]. Increased MYCL gene copies were correlated with the presence of metastases in HNSCC [50]. SET is a serine/threonine phosphatase involved in the regulation of cell proliferation, differentiation, and transformation, which affect necrosis, cisplatin sensitivity and lymph node metastasis of HNSCC [51].

The reason why these specific 6 genes (CRLF2, HSP90AA1, MAP2K1, PAFAH1B2, MYCL and SET) were chosen as prognostic predictors needs to be explained. These genes were selected by an initial screening for cancer driver genes associated with overall survival, followed by LASSO regression and multivariate Cox modeling. The involvement of these genes in HNSCC pathogenesis is supported by prior evidence. For instance, CRLF2 upregulation promotes tumor progression, while HSP90AA1 is linked to chemoresistance. MAP2K1 activation enables proliferation and survival of cancer cells. PAFAH1B2 enhances aggressiveness and MYCL regulates hallmarks of cancer including proliferation and immune evasion. Finally, SET methyltransferase activity modulates tumor microenvironment interactions. By influencing key oncogenic pathways and processes in HNSCC, expression changes in these genes likely contribute to disease progression and impact prognosis. Their combined expression pattern as a multi-gene signature demonstrates robustness for risk-stratification, potentially by capturing distinct complementary aspects of tumor biology associated with survival outcomes in HNSCC patients.

Combining the selected clinical variable age and stage, the APMHO score was generated and showed good discriminative ability for predicting overall survival outcomes in HNSCC, although the 5-year AUC value for the TCGA dataset was lower than that for GSE37642 data. Perineural invasion was significantly associated with the APMHO score, however, it was not significant in the regression analysis and not selected as a component of the APMHO score. The mutational landscape associated with the APMHO score showed TP53 and TTN mutation rates were high in both groups. Recent research reported that TP53 mutation frequency is significantly lower in metastases compared to primary HNSCC [52]. TP53 mutations are associated with higher TMB scores in metastatic tumors and poorer response to immune checkpoint inhibitors [53]. TTN mutation has been correlated with longer progression-free survival and response to immune checkpoint receptor blockade therapy in solid tumors [54] and is also reported to predict a poor prognosis in patients with thyroid cancer [55]. The detailed role of TTN mutation in subtypes of HNSCC is not well understood.

The high APMHO subgroup showed enrichment in several tumor-associated pathways including hypoxia, unfolded protein response, mTORC1 and Glycolysis signaling, while the KRAS signaling pathway was relatively downregulated in this subgroup. mTORC1 signaling pathway has emerged as a crucial player in the oncogenesis and development of HNSCC, and a mTORC1-related gene signature was documented as a novel prognostic factor for HNSCC [56], showing higher mTORC1 immunopositivity conferred worse outcomes in older and higher-risk cases [57]. However, the correlation of mTORC1 target pathways with tumor microenvironment and prognosis in HNSCC is unclear. Consistent with our findings, upregulated glycolysis was correlated with tumor progression and immune evasion in patients with HNSCC [58].

The APMHO score was also associated with variations in the immune landscape of HNSCC, which is associated with response to immunotherapy [59]. CD276, CD70, CD86, NPR1 and TNFSF4 showed higher expression in the high APMHO group. CD70 has been found associated with a higher grade of HNSCC differentiation and comprises a target of CAR-T cells [60]. Similarly, CD86 is overexpressed in multiple cancers and associated with worse outcomes [61], while upregulation of the costimulatory molecule TNFS4 is linked to higher chemoresistance [62]. NPR1 has also been reported as a prognostic marker of worse overall survival in HNSCC [63]. Collectively, these findings may suggest higher immune evasion in the high APMHO subgroup, as CD276 [64] and other highly expressed immune markers may inhibit immune surveillance, and thus this group may benefit from checkpoint blockade. Notably, Liu et al. reported that mTORC1 upregulates B7-H3/CD276 to inhibit antitumor T cells and drive tumor immune evasion [65]. This is consistent with our finding of activation of the mTORC1 signaling pathway and high CD276 expression in high APMHO. IDO2, which showed greater expression in the low AMPHO subgroup, is closely related to tryptophan catabolizing enzymes that have immunomodulatory properties [66]. It has an important role as a modulator of B cell function and its downregulation is implicated as a marker of immune escape [67]. The APMHO score was associated with opposite patterns of association with CD8 memory T cells and activated CD8 T cells. HNSCC is typically rich in CD8 T cell infiltrate but shows poor outcomes [68] and in HNSCC carcinogenesis, a population shift from naïve to memory T cell subtype has been noted [69], aligned with the finding of relative memory CD8 T cell enrichment in the high AMPHO group. CD8 T cell activation is associated with improved prognosis after the recurrence of HNSCC [70, 71]. Of note, CD8 activation marker CD38 positive CD8 T cells have been associated with worse prognosis in HNSCC but also response to PD1 blockade [72]. These findings further support the relevance of AMPHO for precision immunotherapy. The low APMHO group showed significantly higher IPS-PD1/PDL1PDL2 blocker scores suggestive of higher tumor immunogenicity in this subgroup, consistent with greater CD8 T cell activation. Furthermore, the interferon-γ pathway markers and m6A regulators also showed different expression patterns associated with APMHO score profiles. Interferon receptor signaling promotes cancer stemness and effector CD8 + T-cell exhaustion and activating Interferon-γ signaling is associated with poor immunogenicity and worse clinical outcomes in HNSCC [72]. m6A methylation plays an important role in tumor immune microenvironment regulation and response to immune checkpoint inhibitors. m6A methylation pattern represented by a higher level of FTO was earlier found to predict improved prognosis in HNSCC [73], which was consistent with our finding of high expression in high APMHO. These findings suggested that APMHO scores were significantly related to tumor immune environment and cancer immunotherapy-related genes. Further studies are needed to understand the response patterns to immune checkpoint blockers concerning APMHO score and precision immunotherapy.

Among the clinical variables used to design the AMPHO, age, gender, and stage of HNSCC were utilized to leverage the most commonly documented independent clinical variables in comprehensive prognostic scoring to achieve good accuracy. In addition, in a 10-year survival analysis, increasing age and tumor stage were documented as significant clinical factors impacting overall survival [74], while gender has been found to significantly impact HNSCC overall survival and have significant interaction with race [75]. Future model development could include additional clinical and molecular variables and evaluate the optimal selection of predictors. A key limitation of the present study is that the validation dataset in this model included HPV-negative HNSCC cases [76], which comprises only one phenotype of HNSCC. Molecular subtyping of HNSCC has identified several subtypes among which mesenchymal and hypoxia-associated subtypes were found to be more aggressive [77]. The incorporation of multiple validation cohorts with diverse phenotypical and molecular subtypes in the validation process is important for confirming the generalizability and refinement of the AMPHO score.

The development of a robust and validated prognostic model like APMHO can have significant clinical impact for head and neck cancer management. If incorporated into clinical decision making, it can identify patients at high risk of mortality who may benefit from more aggressive treatment or closer monitoring after standard therapy. Conversely, patients classified as low risk by APMHO may be considered for treatment de-escalation to reduce toxicity without compromising outcomes. Beyond prognosis, APMHO may also predict response to emerging immunotherapies as it is associated with differences in immune phenotypes and checkpoint expression. Patients with favorable tumor immune microenvironments may be rational candidates for immunotherapy based on their APMHO scores. Finally, the genes and pathways associated with APMHO risk groups can reveal biological insights and therapeutic targets relevant to high risk disease. In summary, the clinical translation of a well-validated prognostic model like APMHO has the potential to enhance risk stratification, inform treatment decisions, and guide new therapeutic approaches for improved outcomes in head and neck cancer patients. However, further validation in diverse clinical cohorts is needed to realize these impacts.

This study has several limitations that warrant acknowledgement. Firstly, the lack of experimental validation of the findings remains a key limitation, as computational discoveries may not fully recapitulate biological complexity. Some preliminary lab-based validation experiments should be planned to lend more credibility to the findings. One approach could be to evaluate the expression levels of the identified prognostic genes (e.g. CRLF2, HSP90AA1, MAP2K1 etc.) in a small set of clinical HNSCC samples using RT-qPCR. Another approach could be to treat HNSCC cell line models or patient-derived xenografts with immunotherapy agents and evaluate if prognostic gene signatures predict treatment response. Secondly, validation was limited to only one independent dataset, which restricts generalizability of the APMHO model across diverse HNSCC cohorts and molecular subtypes. One approach for validation could be to develop APMHO as a multi-gene assay and evaluate its prognostic performance in additional and multiple independent large HNSCC cohorts to rigorously confirm its prognostic utility. Thirdly, the retrospective design and use of public data carries inherent biases and batch effects that may impact analysis outcomes. Prospective clinical trials are imperative to test the APMHO model’s real-world predictive performance. Fourthly, the current study did not seem to control for or account for important potential confounding factors like environmental exposures, lifestyle choices such as smoking and alcohol consumption that are major risk factors for HNSCC, co-morbidities, and quality of care received by the patients. The prognostic model developed in the present research could be biased without adjusting for these confounders that can independently influence survival outcomes in HNSCC. Finally, the specific mechanisms underlying the associations between prognostic gene/pathway alterations and survival outcomes remain to be fully elucidated. Detailed functional studies are necessary to uncover these mechanistic links. In summary, while promising, the current findings are preliminary in nature due to the lack of robust experimental and clinical validation along with mechanistic investigation, which are essential next steps to establish the utility of this prognostic gene signature and integrated predictive model in HNSCC.

The potential impact and implication of these findings in the oncology field needs to be emphasized. This study demonstrates the benefit of integrating multi-modal data into a robust prognostic model for personalized risk assessment in cancer, with important implications for precision oncology. By combining transcriptomic profiles and clinical variables, the APMHO score provides more accurate survival prediction in head and neck squamous cell carcinoma compared to using either dataset alone. If clinically validated, such integrated “omics-clinical” prognostic tests could significantly improve individualized risk stratification to guide management and treatment decisions. Patients stratified as high-risk by APMHO or similar scores may warrant more aggressive therapies or supportive care, while low-risk scores could promote active surveillance. Furthermore, the biological associations found here suggest these models may predict therapeutic response, enabling personalized treatment selection. This work highlights the potential of transcriptomic-clinical integration to capture diverse aspects of tumor heterogeneity within a singular prognostic tool to transform patient care. With further research, scores like APMHO may be adapted to various cancers to guide precision medicine and improve patient outcomes. Overall, this study provides a framework for developing more reliable, personalized prognostic tests by integrating complementary datasets, with profound implications for prognostication and tailored therapy in oncology.

Overall, the present study demonstrated that the constructed APMHO scoring showed good prognostic value for HNSCC and was consistently associated with variations in clinicopathological, mutational, functional, immune cell landscapes, and cancer immunotherapy-related gene expression patterns. These findings are limited by the lack of experimental validation in an independent clinical cohort and batch effects inherent in multi-cohort analyses. The present findings should be considered as a basis for further investigation towards its validity in utility including those in diverse clinical and molecular subgroups of HNSCC.

### Supplementary Information

Additional file 1: Identification of hub genes via LASSO cox regression analysis. (A) LASSO coefficient profiles of the key genes; (B) Partial likelihood deviance of OS for the LASSO coefficient profiles.

Additional file 2: Genes significantly associated with overall survival.

Additional file 3: Genes in both datasets as main effectors.

Additional file 4: Gene pairs with strong reciprocal relationships.

Additional file 5: Gene pairs with strong interactions in both datasets.

Additional file 6: Gene mutation rates between the high and low APMHO groups.

## Data Availability

Data will be provided on reasonable request.

## Abbreviations

HNSCC: Head and neck squamous cell carcinoma
TCGA: The Cancer Genome Atlas
KM: Kaplan–Meier
ROC: Receiver operating characteristic
AUC: Area under the curve
APMHO: Accurate Prediction Model of HNSCC Overall Survival
HPV: Human papillomavirus
CRLF2: Cytokine receptor-like factor 2
HSP90AA1: Heat shock protein 90 alpha family class A member 1
MAP2K1: Mitogen-activated protein kinase kinase 1
PAFAH1B2: Platelet activating factor acetylhydrolase 1b catalytic subunit 2
MYCL: MYC proto-oncogene, bHLH transcription factor
SET: SET nuclear proto-oncogene
DAXX: Death domain associated protein
FLT3: FMS related tyrosine kinase 3
FLT3L: FMS related tyrosine kinase 3 ligand
CCR7: C–C motif chemokine receptor 7
CD79A: Cluster of differentiation 79A
KAT6A: Lysine acetyltransferase 6A
IKBKB: Inhibitor of nuclear factor kappa B kinase subunit beta
ssGSEA: Single sample gene set enrichment analysis
TIDE: Tumor immune dysfunction and exclusion
IPS: Immunophenoscore
RT-qPCR: Reverse transcription quantitative polymerase chain reaction;
PD-1: Programmed cell death protein 1
PD-L1: Programmed death ligand 1
CTLA-4: Cytotoxic T-lymphocyte associated protein 4

## Code availability

Code generated or used during the study are available from the corresponding author by request.
