# Supplementary material for: A comprehensive prognostic score for head and neck squamous cancer driver genes and phenotype traits
Source: Discov Oncol. 2023 Oct 28;14:193. doi: 10.1007/s12672-023-00796-y (PMC10613197; doi:10.1007/s12672-023-00796-y)
Supplement: Supplementary file 1 — Additional file 1: Identification of hub genes via LASSO cox regression analysis. (A) LASSO coefficient profiles of the key genes; (B) Partial likelihood deviance of OS for the LASSO coefficient profiles. [file 12672_2023_796_MOESM1_ESM.docx]

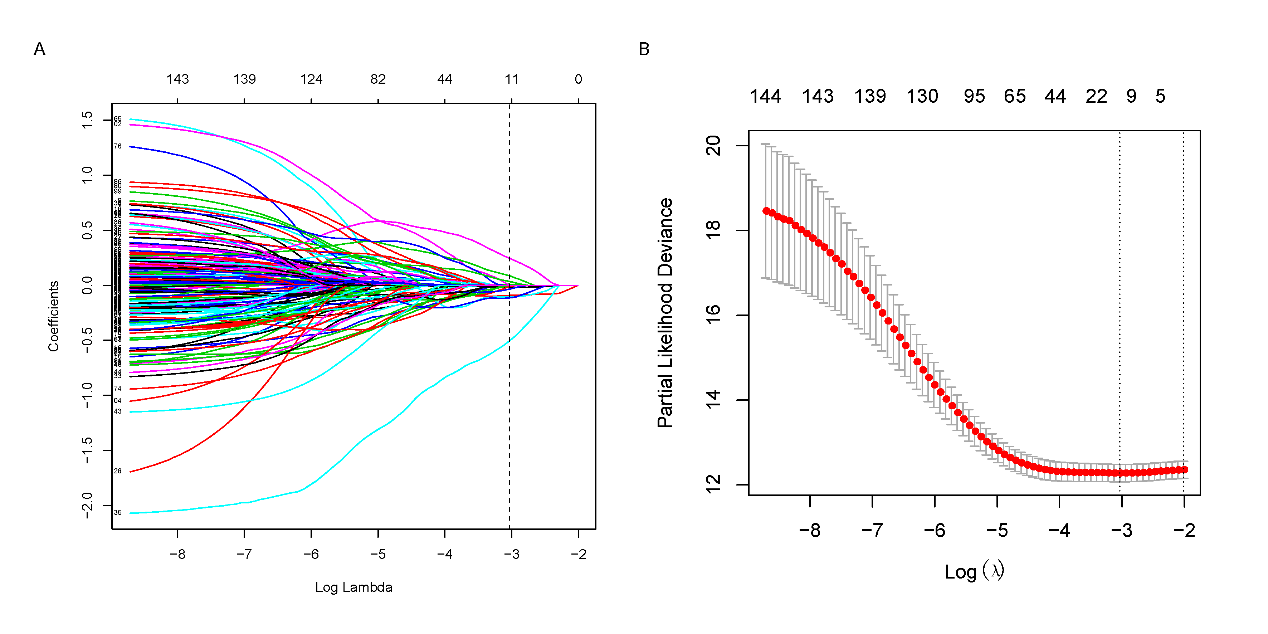


**Supplementary Figure 1. Identification of hub genes via LASSO cox regression analysis. (A) LASSO coefficient profiles of the key genes；(B) Partial likelihood deviance of OS for the LASSO coefficient profiles.**
